# Long-term outcome of patients with severe pulmonary hypertension undergoing transcatheter aortic valve implantation

**DOI:** 10.3389/fcvm.2025.1678025

**Published:** 2026-01-30

**Authors:** Oliver D. Bhadra, Jessica Weimann, Lara Waldschmidt, Till J. Demal, Ina von der Heide, Laura Hannen, David Grundmann, Sebastian Ludwig, Lisa Voigtlaender-Buschmann, Johannes Schirmer, Simon Pecha, Stefan Blankenberg, Hermann Reichenspurner, Moritz Seiffert, Lenard Conradi, Niklas Schofer, Andreas Schaefer

**Affiliations:** 1 Department of Cardiovascular Surgery, University Heart & Vascular Center Hamburg, Hamburg, Germany; 2 Department of Cardiology, University Heart & Vascular Center Hamburg, Hamburg, Germany; 3 Department of Cardiology, BG Universitätsklinikum Bergmannsheil Bochum, Bochum, Germany

**Keywords:** TAVI, SPAP, long term follow up, aortic valve disease, pulmonary hypertension

## Abstract

**Objectives:**

Recent reports suggest that pulmonary hypertension (PH) is associated with a significantly higher acute mortality after transcatheter aortic valve implantation (TAVI). The aim of this study is to characterize patients undergoing TAVI with preoperative echocardiographically determined severe PH and to investigate acute clinical and long-term outcomes.

**Methods:**

From 2008 to 2021, 3,610 patients with preoperatively documented systolic pulmonary artery pressure (sPAP) underwent TAVI at our institution. The cut off for severe PH was defined as sPAP > 55 mmHg as determined by echocardiography. Severe PH was preoperatively identified in 456 patients. This group was compared to 3,154 patients with sPAP ≤ 55 mmHg. Data were retrospectively analysed according to updated Valve Academic Research Consortium (VARC-3) definitions.

**Results:**

TAVI patients with sPAP > 55 mmHg presented with higher median age (sPAP ≤ 55 mmHg: 81.6 years [interquartile range (IQR): 77.2–85.1] vs. sPAP > 55 mmHg: 82.3 (IQR 77.8–85.8), *p* = 0.01) and higher prevalence of significant left ventricular dysfunction (LVEF < 35%) (9.7 vs. 15.5%, *p* < 0.001). Acute outcomes were impaired in patients with severe PH. The detrimental effect of severe PH persisted in Kaplan–Meier analysis one-year after TAVI (mortality rate 20.0 vs. 30.2%, *p* < 0.001) and in 60-month follow-up (52.0 vs. 65.1%, *p* < 0.001).

**Conclusion:**

TAVI patients with severe PH represent a high-risk subgroup with unfavourable acute outcomes and increased one-year and long-term mortality. Moreover, the presence of severe PH is associated with increased rates of acute adverse events, including bleeding, need for PPM implantation and renal failure.

## Introduction

Transcatheter Aortic Valve Implantation (TAVI) has proven clinical safety and efficacy in patients across all risk strata (1–3). Among comorbidities associated with impaired outcomes after TAVI, pulmonary hypertension (PH) represents an independent risk factor for significant acute morbidity and mortality after TAVI as shown in recent reports (4–6). Five mechanisms of PH are classified according to the updated clinical classification (7). PH associated with left heart disease is the most prevalent form (8). Recent reports suggest that functional and structural anomalies of the aortic valve may lead to progressive cardiac downstream impairment including the left ventricle, left atrium, pulmonary vasculature, and eventually, the right heart chambers with consecutive post-capillary pulmonary hypertension (Ipc-PH) or combined pre- and post-capillary pulmonary hypertension (Cpc-PH). Although the presence of PH in patients suffering from severe AS is indicative of a limited prognosis, mainly due to increased rates of sudden cardiac death and accelerated exacerbation of symptoms, the prevalence of PH among patients with AS remains unclear (9–11). This ambiguity may be partly associated with the fact that invasive right heart catheterization is considered the gold standard for evaluation of PH, but its routine use is limited in clinical practice due to the invasive nature and associated risks (12).

Therefore, non-invasive assessment of PH using echocardiography has become the preferred method in TAVI patients. Echocardiography provides reliable information on systolic pulmonary artery pressure (sPAP), which serves as a surrogate marker for PH severity (13). Previous studies have demonstrated the utility of sPAP in assessing PH and its role in predicting clinical outcomes in TAVI patients (13, 14). However, long-term outcomes of patients undergoing TAVI with preoperative significant PH are not investigated so far.

In this work, we aim to present our single-center experience of patients with echocardiographically determined PH undergoing TAVI and report long-term outcomes of this special subset of patients.

## Methods

### Patients

This retrospective analysis included a total of 3,610 patients who underwent TAVI between 03/2008 and 01/2023 at our center. Allocation of patients to TAVI followed current international recommendations after consensus of the local dedicated heart team. Due to the retrospective study design and anonymous data collection, written patients informed consent was waived as reflected and approved by our local ethical committee.

### Data collection, diagnostic work-up and study procedure

Baseline characteristics, procedural data, and outcomes were collected and retrospectively analyzed. Preoperative echocardiographic data were specifically examined to assess PH in the patient population. The classification of PH was determined using a semi-quantitative approach based on sPAP measured during echocardiography. A threshold of 55 mmHg was used to distinguish between significant and non-significant PH as proposed by the EuroSCORE II (15, 16). Pulmonary hypertension is therefore divided into two categories according to the EuroSCORE II: >55 mmHg and 55–31 mmHg (16). Current institutional standard proceedings for TAVI procedures were applied as described before for patients treated over the last 5 years (17, 18). Due to the large investigated time frame substantial procedural changes occurred during the study period (e.g., general anesthesia vs. local anesthesia, femoral secondary access vs. radial secondary access) Clinical endpoints were adjudicated in accordance with the updated standardized Valve Academic Research Consortium (VARC) 3 criteria (19). The median follow-up time was 3 years.

### Statistical analysis

Continuous variables are shown as medians (25th percentile, 75th percentile) and compared using the Mann–Whitney *U*-test. Binary variables are shown as counts (frequencies) and compared using the *χ*^2^ test.

The median follow-up time was estimated by the reverse Kaplan–Meier estimator. To assess differences in mortality between patients with lower vs. elevated sPAP, survival probabilities of patients were estimated using the Kaplan–Meier method. Groups are compared using the log-rank test.

Multivariable regression analysis was conducted to examine the association between sPAP and various outcomes adjusted by age, log transformed STS risk score, LVEF categories, and transapical access vs. other. Results are shown as Odds Ratios (OR) for logistic regression and as Hazard Ratios (HR) for Cox regression analysis, in case of mortality endpoint, with corresponding 95% confidence intervals (CI). Left ventricular ejection fraction (LVEF) was entered into the multivariable Cox regression model as a categorical variable with four levels (normal, mildly, moderately and severely reduced LVEF), using normal LVEF as the reference category. A *p*-value of <0.05 was considered statistically significant. All analyses were performed with R statistical software version 4.0.3 (R Foundation for Statistical Computing, Vienna, Austria).

## Results

### Baseline demographics

Of 3,610 patients 3,154 presented a preoperative sPAP ≤ 55 mmHg (group 1) and 456 patients presented with sPAP > 55 mmHg (group 2). Differences in baseline characteristics included a higher median age (group 1: 81.6 [interquartile range (IQR) 77.2–85.1] vs. group 2: 82.3 (IQR 77.8–85.8) years, *p* = 0.014) and risk profile according to common risk stratification tools in group 2. Furthermore, group 2 demonstrated a higher prevalence of severe left ventricular dysfunction (9.7 vs. 15.5%, *p* < 0.001) and more frequently a significantly reduced right ventricular function as reflected by a lower tricuspid annular plane systolic excursion (median 20.0 vs. 17.0 mm, *p* < 0.001). In addition to cardiac dysfunction, patients with a sPAP > 55 mmHg presented a higher prevalence of peripheral artery disease (23.2 vs. 29.6%, *p* = 0.003) and higher median baseline creatinine levels [1.1 (IQR 0.9–1.4) vs. 1.2 (IQR 0.9–1.6) vs. mg/dl], *p* < 0.001).

Detailed patient demographics of both groups are summarized in Table 1.

**Table 1:** Baseline demographics.

	All (*N* = 3,610)	≤55 mmHg (*N* = 3,154)	>55 mmHg (*N* = 456)	*p*-value
Age (years)	81.6 (77.3, 85.2)	81.6 (77.2, 85.1)	82.3 (77.8, 85.8)	0.014
Male Gender, *n* (%)	1,848 (51.2)	1,625 (51.6)	223 (49.0)	0.33
EuroSCORE II (%)	4.6 (2.5, 8.2)	4.3 (2.3, 7.9)	6.2 (3.8, 10.5)	<0.001
STS-Risk Score (%)	4.0 (2.5, 6.3)	3.7 (2.3, 6.0)	5.4 (3.6, 8.1)	<0.001
Normal LV function, *n* (%)	2,184 (63.3)	1,947 (64.8)	237 (53.1)	<0.001
Mild LV dysfunction, *n* (%)	487 (14.1)	418 (13.9)	69 (15.5)	0.41
Moderate LV dysfunction, *n* (%)	421 (12.2)	350 (11.6)	71 (15.9)	0.008
Severe LV dysfunction, *n* (%)	360 (10.4)	291 (9.7)	69 (15.5)	<0.001
Arterial hypertension, *n* (%)	2,927 (81.6)	2,546 (81.2)	381 (83.9)	0.16
Diabetes mellitus, *n* (%)	974 (27.1)	855 (27.3)	119 (26.2)	0.67
Coronary artery disease, *n* (%)	2,232 (63.2)	1,959 (63.5)	273 (61.2)	0.38
Peripheral arterial disease, *n* (%)	863 (24.0)	728 (23.2)	135 (29.6)	0.005
Prior stroke, *n* (%)	521 (14.5)	454 (14.4)	67 (14.7)	0.89
COPD, *n* (%)	571 (15.9)	496 (15.8)	75 (16.4)	0.74
Creatinine (mg/dl)	1.1 (0.9, 1.4)	1.1 (0.9, 1.4)	1.2 (0.9, 1.6)	<0.001
Any malignant disease, *n* (%)	765 (21.2)	667 (21.2)	98 (21.5)	0.91
Prior cardiac surgery, *n* (%)	590 (16.5)	506 (16.2)	84 (18.6)	0.23
Mean transvalvular gradient (mmHg)	32.0 (23.0, 44.0)	32.0 (23.0, 44.0)	33.0 (21.0, 45.5)	0.91
Effective orifice area (cm^2^)	0.8 (0.6, 0.9)	0.8 (0.6, 0.9)	0.7 (0.6, 0.9)	<0.001
TAPSE (mm)	19.0 (16.0, 23.0)	20.0 (16.0, 23.0)	17.0 (13.0, 20.4)	<0.001

EuroSCORE, European system for cardiac operative risk evaluation; STS-PROM, society of thoracic surgeons–predicted risk of mortality; LV, left ventricle; COPD, chronic obstructive pulmonal disease; TAPSE, tricuspid annular plane systolic excursion.

### Periprocedural data

A higher proportion of patients with sPAP > 55 mmHg underwent transapical access for TAVI compared to the group with sPAP ≤ 55 mmHg (16.1% vs. 24.6%). Overall, there was a significant difference in the choice of access route between the groups (*p* < 0.001). Furthermore, differences in the selection of prosthetic valves and valve sizes were found. Rates of pre- (74.9% vs. 74%, *p* = 0.73) and post-dilatation (37.0% vs. 33.6%, *p* = 0.16) showed no differences between groups and embolic protection devices were more frequently used in group 2 (8.0% vs. 13.4%, *p* < 0.001).

Detailed periprocedural data are summarized in Table 2.

**Table 2:** Periprocedural data.

	All (*N* = 3,610)	≤55 mmHg (*N* = 3,154)	>55 mmHg (*N* = 456)	*p*-value
Access				0.0010
Other, *n* (%)	4 (0.1)	4 (0.1)	0 (0)	
Transaortic, *n* (%)	9 (0.3)	6 (0.2)	3 (0.7)	
Transapical, *n* (%)	608 (17.1)	497 (16.1)	111 (24.6)	
Transaxillary, *n* (%)	60 (1.7)	48 (1.6)	12 (2.7)	
Transfemoral, *n* (%)	2,865 (80.8)	2,539 (82.1)	326 (72.1)	
Implanted THV				<0.001
Sapien XT/Sapien3/Sapien3 Ultra, *n* (%)	1,507 (42.4)	1,318 (42.4)	189 (43.8)	
CoreValve/Evolut/Evolut R, *n* (%)	873 (24.6)	794 (25.5)	79 (17.5)	
Acurate, *n* (%)	577 (16.2)	496 (16.0)	81 (17.9)	
JenaValve, *n* (%)	146 (4.1)	113 (3.6)	33 (7.3)	
Portico/Navitor, *n* (%)	143 (4.0)	124 (4.0)	19 (4.2)	
Lotus, *n* (%)	79 (2.2)	59 (1.9)	20 (4.4)	
Engager, *n* (%)	73 (2.1)	63 (2.0)	10 (2.2)	
Allegra, *n* (%)	57 (1.6)	39 (1.3)	18 (4.0)	
Other, *n* (%)	66 (1.9)	66 (2.1)	0 (0)	
Centera, *n* (%)	20 (0.6)	18 (0.6)	2 (0.4)	
Biovalve, *n* (%)	14 (0.4)	13 (0.4)	1 (0.2)	
Prosthetic valve size (mm)	26.0 (25.0, 29.0)	26.0 (25.0, 29.0)	26.0 (23.0, 27.0)	0.017
Pre-dilatation, *n* (%)	2,635 (74.8)	2,304 (74.9)	331 (74.0)	0.73
Post-dilatation, *n* (%)	1,266 (36.5)	1,117 (37.0)	149 (33.6)	0.16
Use of cerebral protection device, *n* (%)	304 (8.7)	244 (8.0)	60 (13.4)	<0.001
Procedure time (minutes)	80.0 (60.0, 100.0)	80.0 (60.0, 100.0)	80.0 (65.0, 105.0)	0.013
Contrast amount (mL)	160.0 (120.0, 205.0)	159.5 (120.0, 204.0)	160.0 (120.0, 210.0)	0.50

THV, transcatheter heart valve.

### Echocardiographic and clinical outcome at 30 days

**Figure 1:**
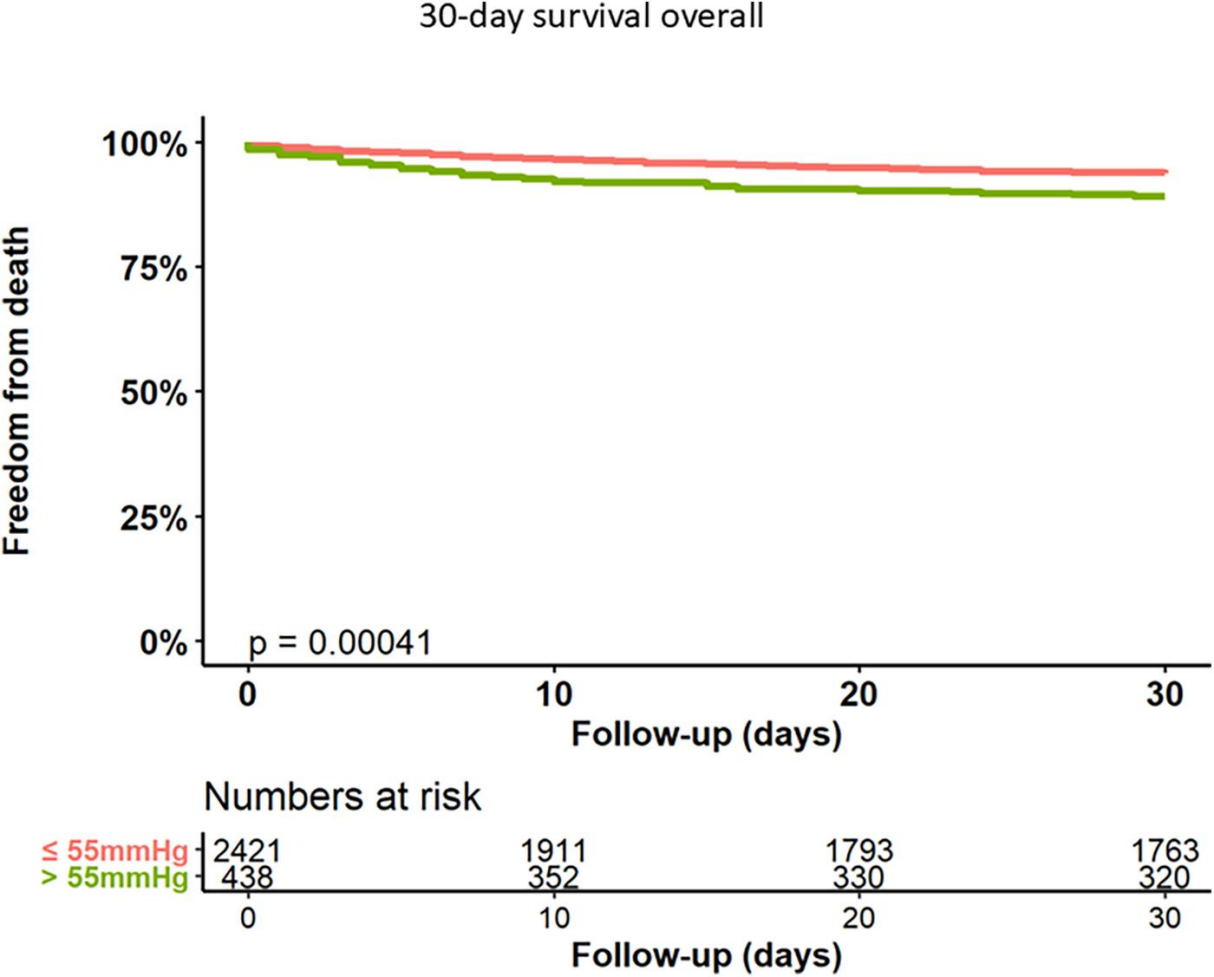
Kaplan–Meier analysis of 30-day follow-up for freedom from death.

Rates of acute kidney injury [according to Acute Kidney Injury Network (AKIN) stage II or III] were higher in group 2 (3.2% vs. 7.0%, *p* < 0.001). Patients with sPAP > 55 mmHg exhibited higher rates of severe or life-threatening bleeding (8.5% vs. 15%, *p* < 0.001) and severe paravalvular regurgitation (3.4% vs. 5.7%, *p* = 0.028) compared to group 1. Moreover, the 30-day mortality rate was higher in patients with severe PH (6.4% vs. 11.0%, log rank test *p* < 0.001). Kaplan Meyer curve for 30-day mortality is shown in Figure 1.

Detailed outcome parameters are documented in Table 3.

**Table 3:** Clinical events.

	All (*N* = 3,610)	≤55 mmHg (*N* = 3,154)	>55 mmHg (*N* = 456)	*p*-value
Valve malposition, *n* (%)	62 (2.7)	57 (3.0)	5 (1.2)	0.042
Cardiac tamponade, *n* (%)	18 (0.8)	15 (0.8)	3 (0.7)	1.00
Coronary ostia occlusion, *n* (%)	1 (0.0)	1 (0.1)	0 (0)	1.00
Root rupture, *n* (%)	10 (0.4)	9 (0.5)	1 (0.2)	0.69
Severe paravalvular regurgitation, *n* (%)	123 (3.7)	100 (3.4)	23 (5.7)	0.028
Non-disabling stroke, *n* (%)	38 (1.4)	31 (1.3)	7 (1.6)	0.84
Disabling stroke, *n* (%)	62 (2.2)	50 (2.2)	12 (2.7)	0.49
Length of ICU stay (days)	1.0 (1.0, 2.0)	1.0 (1.0, 2.0)	1.0 (1.0, 2.0)	<0.001
Major vascular complication, *n* (%)	236 (7.9)	192 (7.5)	44 (10.0)	0.076
Major or life-threatening bleeding, *n* (%)	283 (9.5)	217 (8.5)	66 (15.0)	<0.001
Permanent pacemaker implantation, *n* (%)	436 (14.6)	357 (14.0)	79 (18.0)	0.042
Acute kidney injury stage II or III, *n* (%)	132 (3.7)	100 (3.2)	32 (7.0)	0.001
Myocardial infarction, *n* (%)	30 (1.0)	24 (0.9)	6 (1.4)	0.43
Postprocedural mean gradient (mmHg)	8.0 (6.0, 12.0)	8.0 (6.0, 12.0)	8.0 (6.0, 12.0)	0.89
VARC-3 device success, *n* (%)	2,746 (91.8)	2,338 (91.7)	408 (92.1)	0.85
VARC-3 early safety, *n* (%)	739 (28.6)	623 (29.0)	116 (26.2)	0.25
30-day mortality, *n* (%)	181 (7.1)	136 (6.4)	45 (11.0)	0.001
1-year mortality, *n* (%)	495 (21.6)	383 (20.0)	112 (30.2)	<0.001

ICU, intensive care unit; VARC, valve academic research consortium 3.

**Figure 2:**
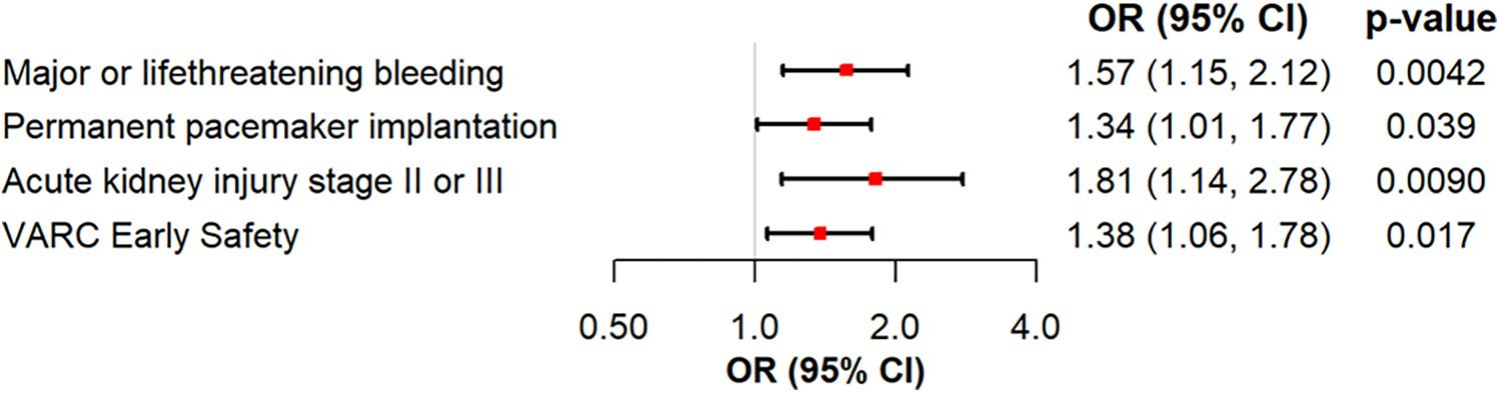
Logistic regression analysis of severe PH for major or life-threatening bleeding, permanent pacemaker implantation, acute kidney injury stage II or III and VARC early safety.

Logistic regression analyses were performed to further investigate the influence of severe PH, age, log transformed STS-Risk Score, transapical access vs. others, and left ventricular function on clinical endpoints**.** An sPAP > 55 mmHg was found to be a predictor for severe and life-threatening bleeding events (OR 1.58, 95% CI 1.15–2.13, *p* = 0.0037). Furthermore, logistic regression analysis demonstrated that sPAP > 55 mmHg is predictive for permanent pacemaker (PPM) implantation (OR 1.34, 95% CI 1.01–1.76, *p* = 0.04), severe kidney injury (AKIN II/III) (OR 1.93, 95% CI 1.23–2.95, *p* = 0.003), and absence of the VARC early safety endpoint (OR 1.38, 95% CI 1.06–1.79, *p* = 0.015) as shown in Figure 2. The logistic regression analyses for the effects of the adjustment variables are shown in Supplementary Figures S1a–d.

### Long-term outcomes

**Figure 3:**
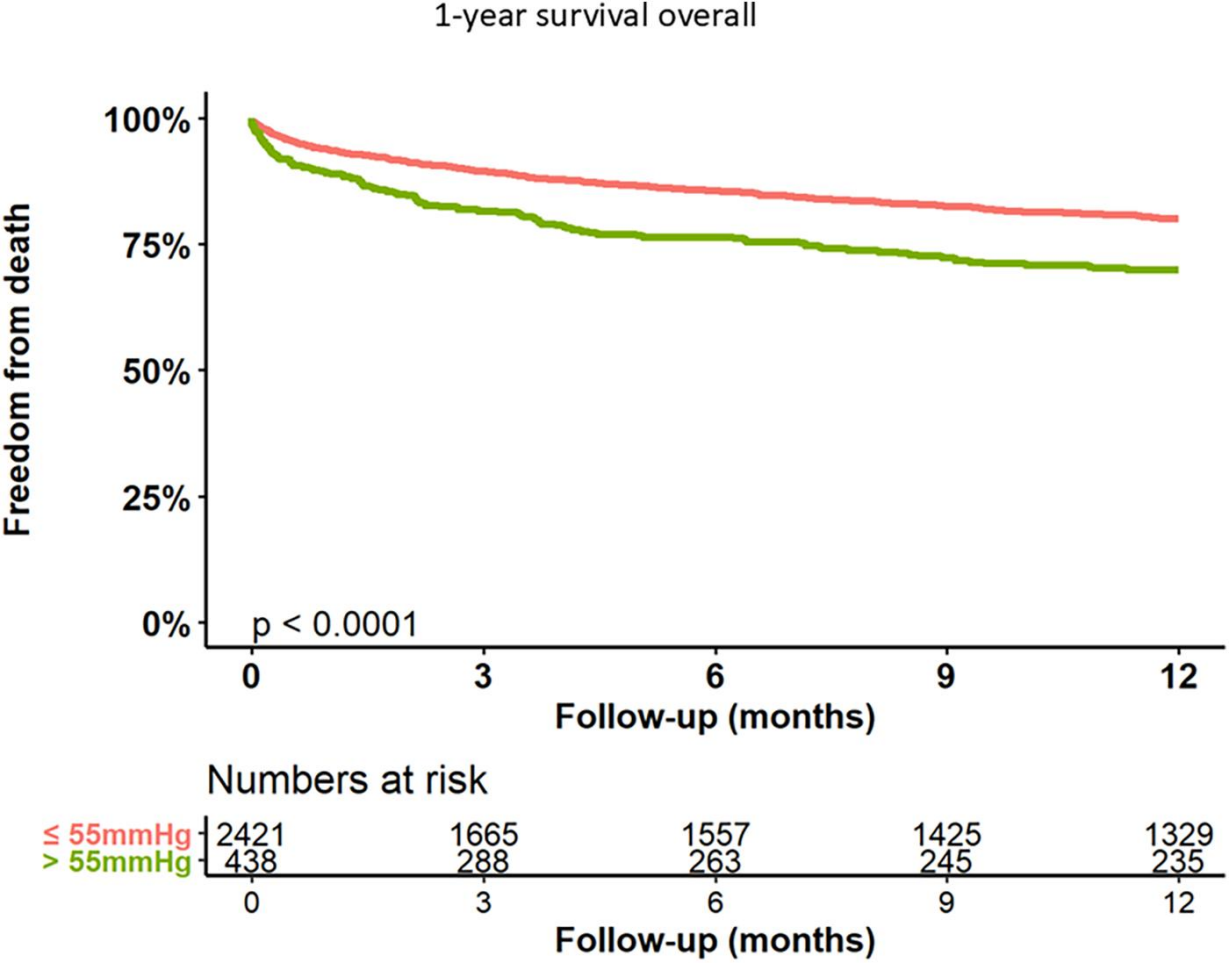
Kaplan–Meier analysis of 12-months follow-up for freedom form death.

**Figure 4:**
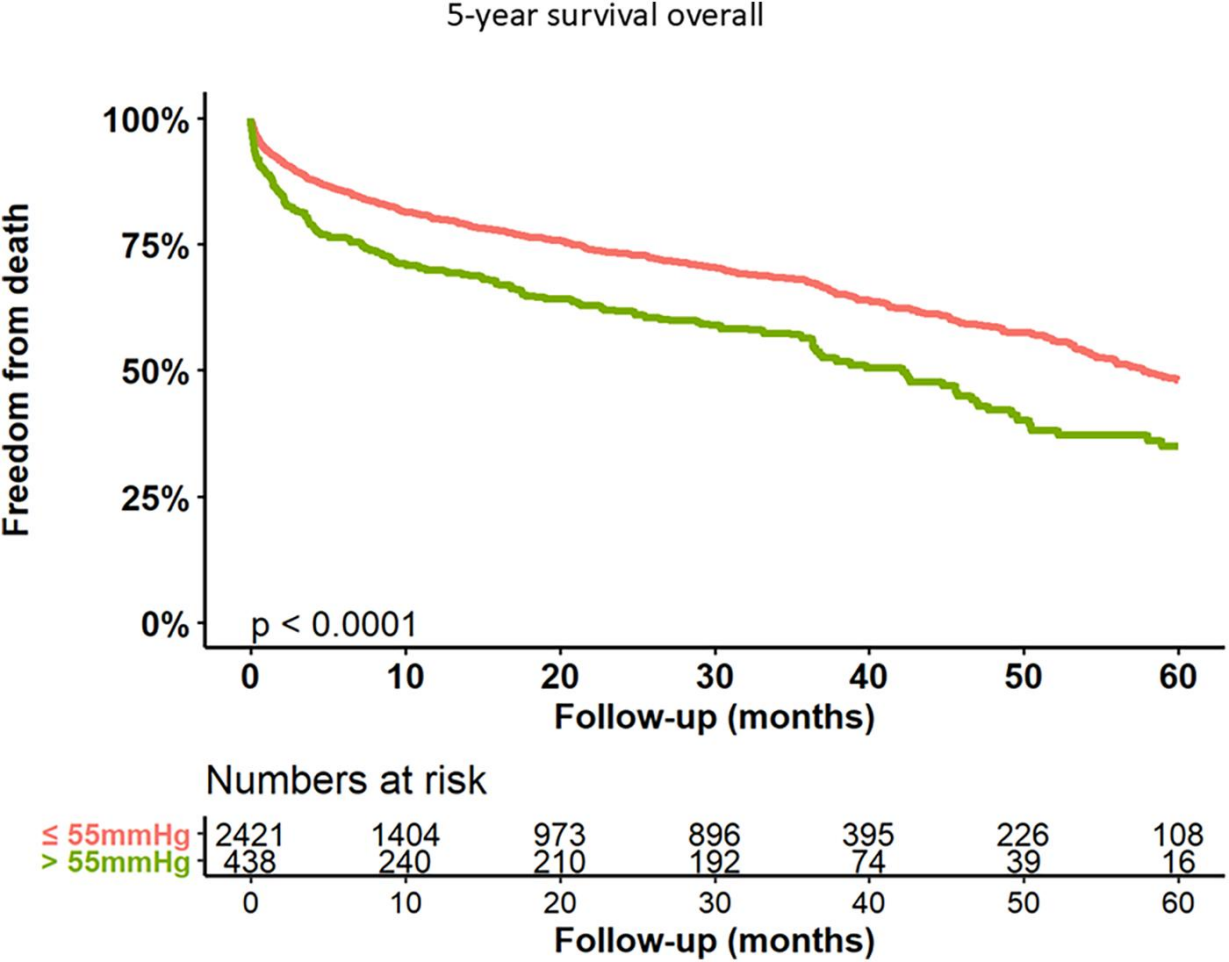
Kaplan–Meier analysis of 60-months follow-up for freedom form death.

**Figure 5:**
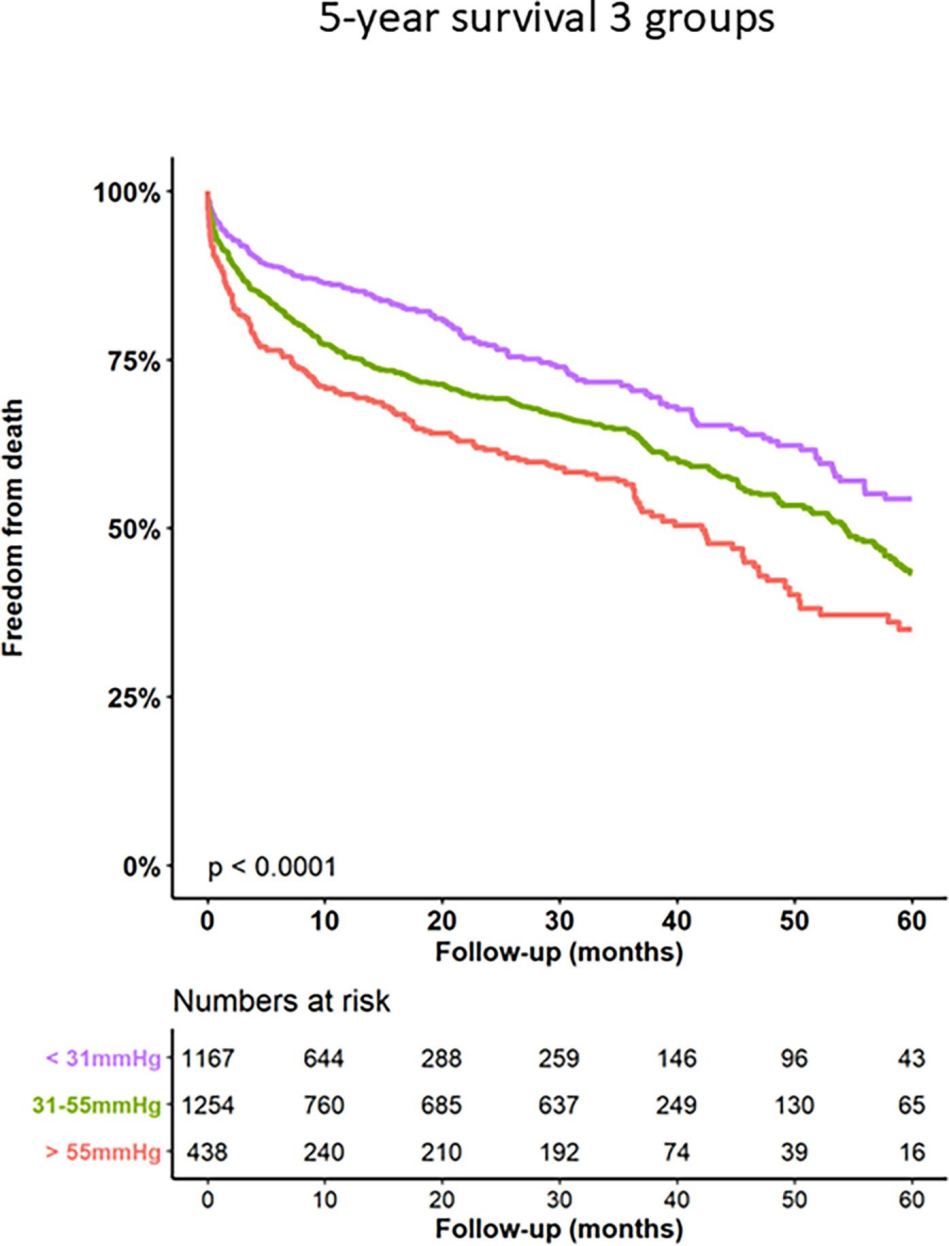
Kaplan–Meier analysis of 60-months follow-up for freedom form death for 3 sPAP subgroups.

**Figure 6:**
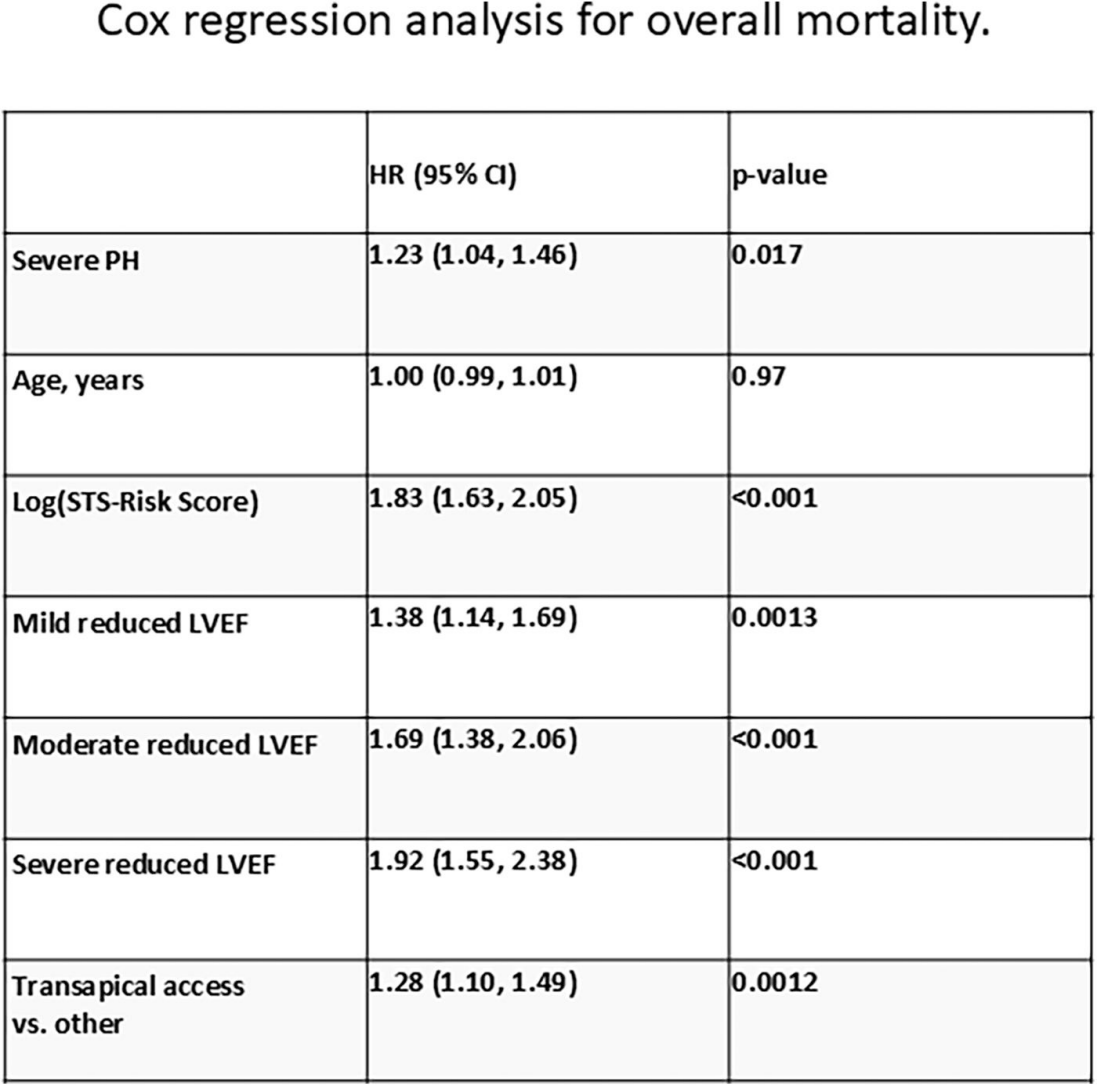
Cox regression analysis for overall mortality.

Kaplan–Meier curves for freedom of death up to 12 months are presented in Figure 3 and long-term outcomes up to 60 months of both groups in Figure 4. Here, a significant detrimental effect of severe PH on mortality over long-term follow-up is illustrated. The one-year mortality rate was higher in group 2 (20.0 vs. 30.2%, *p* < 0.001). At 60-month follow-up, a significantly higher mortality in patients with sPAP > 55 mmHg was seen (52.0% vs. 65.1%, *p* < 0.001). Furthermore, we performed subanalysis comparing the survival of three groups divided according to the sPAP: <31 mmHg vs. 31–55 mmHg vs. >55 mmHg. Here, there was a significant deterioration in 5-year survival with an increase in sPAP in each of the three groups (Figure 5). Cox regression analysis in Figure 6 showed that severe PH is associated with overall mortality (HR 1.23, 95% CI 1.04–1.46, *p* = 0.017). Additionally, an analysis was exclusively conducted on patients who underwent treatment through a transfemoral approach. 2,539 patients with sPAP ≤ 55 mmHg and 326 patients with sPAP > 55 mmHg were included. The 60-month mortality was still significantly higher in patients with sPAP > 55 mmHg (50.5% vs. 56.1%, *p* < 0.001) Supplementary Figure S2. We also analyzed the 1-year survival for 3 different time periods (2008–2014 vs. 2014–2019 vs. 2019–2023). This showed a significantly lower 1-year survival in the group with sPAP > 55 mmHg for the period 2008–2014 and 2019–2023 Supplementary Figures S3a–c.

## Discussion

The main findings of the herein conducted study are:

1. TAVI patients with severe pulmonary hypertension (PH), as indicated by sPAP > 55 mmHg, represent a high-risk subgroup with higher age, a higher surgical risk profile and higher frequencies of left and right ventricular dysfunction.
2. Patients with severe PH undergoing TAVI demonstrate increased periprocedural complication rates including higher incidence of acute kidney injury, severe or life-threatening bleeding, PPM implantation and adverse 30-day, 1-year, and long-term mortality rates up to 60 months.
3. Logistic regression analyses showed that severe PH is a predictor for life-threatening bleeding, PPM implantation, severe kidney injury, and the absence of VARC early safety.

The findings of our study are consistent with previous research, which also demonstrated the association between severe PH and adverse acute outcomes and higher mortality in TAVI patients (5, 16, 20–23). Luçon, A. et al. reported a significantly lower 1-year mortality in patients with sPAP < 40 mmHg compared to patients with sPAP 40–59 mmH and ≥60 mmHg (22% vs. 28% vs. 28%). However, in contrast to our study, there was no significant difference in 30-day mortality between those groups (10% vs. 10% vs. 11%) (4). Another multi-center registry from Testa et al. showed that PH is a predictive factor for 1-year mortality after TAVI and that the persistence of severe PH at 1 month after TAVI is a stronger predictor than baseline severe PH (24). In the mentioned study, a mortality rate of 26% was reported in patients with sPAP ≥ 60 mmHg at the 12-month follow-up after TAVI implantation. Miyamoto et al. reported on the prospective OCEAN-TAVI registry with a follow-up of 24 months. Here, increased overall mortality and heart failure hospitalization were observed in patients with severe PH (25). The results of our cohort are in line with outcomes of these mentioned studies and demonstrate that patients with severe PH are a high-risk patient cohort with high acute mortality after TAVI which persists even in the long-term. Our study expands on these findings by providing long-term follow-up data of 60 months from a high-volume single-center experience and incorporating logistic regression analyses to further examine the independent influence of sPAP > 55 mmHg on clinical endpoints. In our cohort patients with severe PH undergoing TAVI demonstrate impaired acute and long-term outcomes as herein shown by prolonged ICU stay, higher incidence of acute kidney injury, severe or life-threatening bleeding, and elevated 30-day, 1-year, and long-term mortality rates up to 60 months. The herein described rather high acute mortality rates compared to contemporary values is clearly attributable to the investigated historical high-risk patient cohort, which reached back to 2008. Logistic regression analyses showed that severe PH is an independent predictor for life-threatening bleeding, PPM implantation, severe kidney injury, and absence of VARC early safety in our study, even with the included variable TA access, which was shown to be predictive for impaired outcomes after TAVI in the past. Differences in outcomes among different studies may result from discordant definitions of severe PH. As mentioned above, we aligned our threshold of sPAP > 55 mmHg with the definition of severe PH of the EuroSCORE.

The variables “age”, “STS-Risk-Score”, “left ventricular function”, and “transapical or other access” were used to adjust the logistic regression model. The herein found correlation of significant bleeding, PPM implantation, acute kidney injury and absence of VARC early safety in patients with severe PH, align with existing guidelines and recommendations for the management of patients with PH undergoing invasive procedures (12). Therefore, these patients should undergo a particular risk assessment before TAVI, in the context of evaluation by the local dedicated heart team. Specific periprocedural measures to avoid the described complications connected with severe PH may include a sophisticated transfusion and hemostasis management, high implantation of THV using cusp-overlap technique to lessen interaction of the valve frame with the conduction system and sparing of contrast agent. Moreover, these patients should be closely followed up by the center of care after discharge. The identification of this association highlights the importance of considering PH severity when evaluating procedural risks, planning postoperative care, and implementing preventive strategies to mitigate these complications.

## Conclusion

Patients with severe PH undergoing TAVI represent a subgroup at high risk. This subgroup is older with an increased surgical risk profile, including more frequent left and right ventricular dysfunction. Outcomes of these patients are impaired during acute and long-term follow-up with increased rates of acute kidney injury, severe or life-threatening bleeding, PPM implantation and a higher mortality rate at 30 days, 1 year, and up to 60 months post-procedure. Logistic regression showed that severe PH is a predictor for critical complications post-TAVI, such as life-threatening bleeding, the necessity for PPM implantation, severe kidney injury, and absence of early safety. Therefore, thorough evaluation of these patients and anticipation of possible complications with implementation of specific periprocedural measures is of paramount importance to improve outcomes in this special subset of patients.

## Limitations

The retrospective nature of the analysis may introduce inherent biases and limit the ability to establish causality. As a single-center experience, the generalizability of our findings to other settings may be subject to variation in patient populations and procedural practices. The use of non-invasive sPAP estimation as a surrogate marker for PH, while also widely used, may not capture the complete hemodynamic profile of PH. Furthermore, the herein conducted analysis comprises of patients treated by TAVI from 2008 till 2023. During the study period, not only procedural changes were implemented but also THV systems were subject to several advancements.

## Data Availability

The datasets generated and analyzed during the current study are not publicly available due to institutional data-sharing policies but are available from the corresponding author upon reasonable request.
