# Supplementary material for: Long-term outcome of patients with severe pulmonary hypertension undergoing transcatheter aortic valve implantation
Source: Front Cardiovasc Med. 2026 Jan 30;12:1678025. doi: 10.3389/fcvm.2025.1678025 (PMC12902943; doi:10.3389/fcvm.2025.1678025)
Supplement: Supplementary file 1 [file Image1.pdf]

Supplementary Figure 1A.

Logistic regression analysis for Major or life-threatening bleeding

	OR (95% CI)	p-value
Severe PH	1.57 (1.15, 2.12)	0.0042
Age, years	0.98 (0.97, 1.00)	0.090
Log(STS-Risk Score)	1.44 (1.19, 1.74)	<0.001
Mild reduced LVEF	0.91 (0.62, 1.31)	0.62
Moderate reduced LVEF	0.90 (0.60, 1.31)	0.60
Severe reduced LVEF	0.90 (0.57, 1.37)	0.62
Transapical access vs. other	1.35 (1.01, 1.79)	0.038

Supplementary Figure 1B.

Logistic regression analysis for Permanent pacemaker implantation

	OR (95% CI)	p-value
Severe PH	1.34 (1.01, 1.77)	0.039
Age, years	1.01 (0.99, 1.03)	0.24
Log(STS-Risk Score)	1.09 (0.92, 1.29)	0.33
Mild reduced LVEF	0.94 (0.68, 1.28)	0.69
Moderate reduced LVEF	0.73 (0.51, 1.03)	0.083
Severe reduced LVEF	0.80 (0.54, 1.17)	0.26
Transapical access vs. other	0.93 (0.71, 1.21)	0.60

Supplementary Figure 1C.

Logistic regression analysis for Acute kidney injury stage II or III

	OR (95% CI)	p-value
Severe PH	1.81 (1.14, 2.78)	0.0090
Age, years	0.99 (0.96, 1.02)	0.50
Log(STS-Risk Score)	1.85 (1.40, 2.41)	<0.001
Mild reduced LVEF	1.06 (0.61, 1.75)	0.83
Moderate reduced LVEF	0.93 (0.50, 1.61)	0.79
Severe reduced LVEF	0.74 (0.35, 1.44)	0.41
Transapical access vs. other	3.26 (2.20, 4.83)	<0.001

Supplementary Figure 1D.

Logistic regression analysis for VARC Early Safety

	OR (95% CI)	p-value
Severe PH	1.38 (1.06, 1.78)	0.017
Age, years	0.99 (0.98, 1.01)	0.33
Log(STS-Risk Score)	1.49 (1.26, 1.77)	<0.001
Mild reduced LVEF	1.09 (0.80, 1.48)	0.58
Moderate reduced LVEF	1.10 (0.80, 1.50)	0.56
Severe reduced LVEF	0.77 (0.51, 1.13)	0.20
Transapical access vs. other	1.10 (0.86, 1.40)	0.44
