# Supplementary figures and images for: Long-term outcome of patients with severe pulmonary hypertension undergoing transcatheter aortic valve implantation
Source: Front Cardiovasc Med. 2026 Jan 30;12:1678025. doi: 10.3389/fcvm.2025.1678025 (PMC12902943; doi:10.3389/fcvm.2025.1678025)

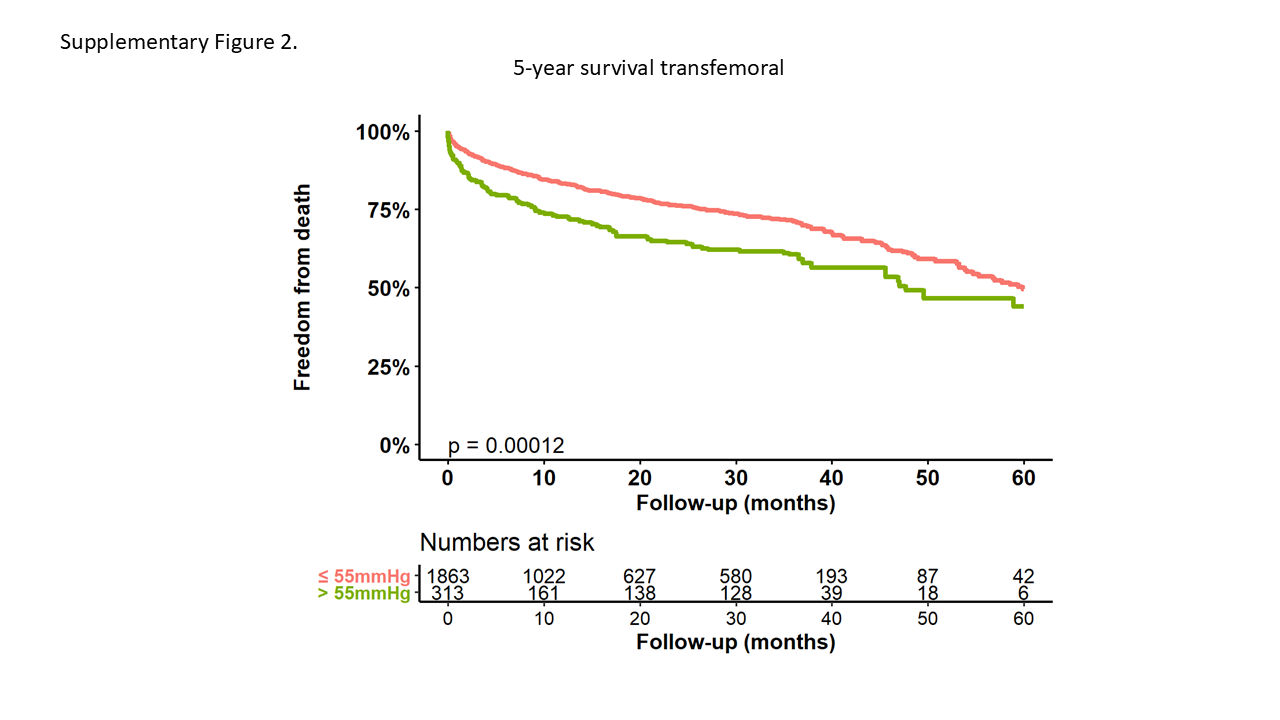

Supplement: Supplementary file 2 [file Image2.tif]

Supplementary Figure 3a.

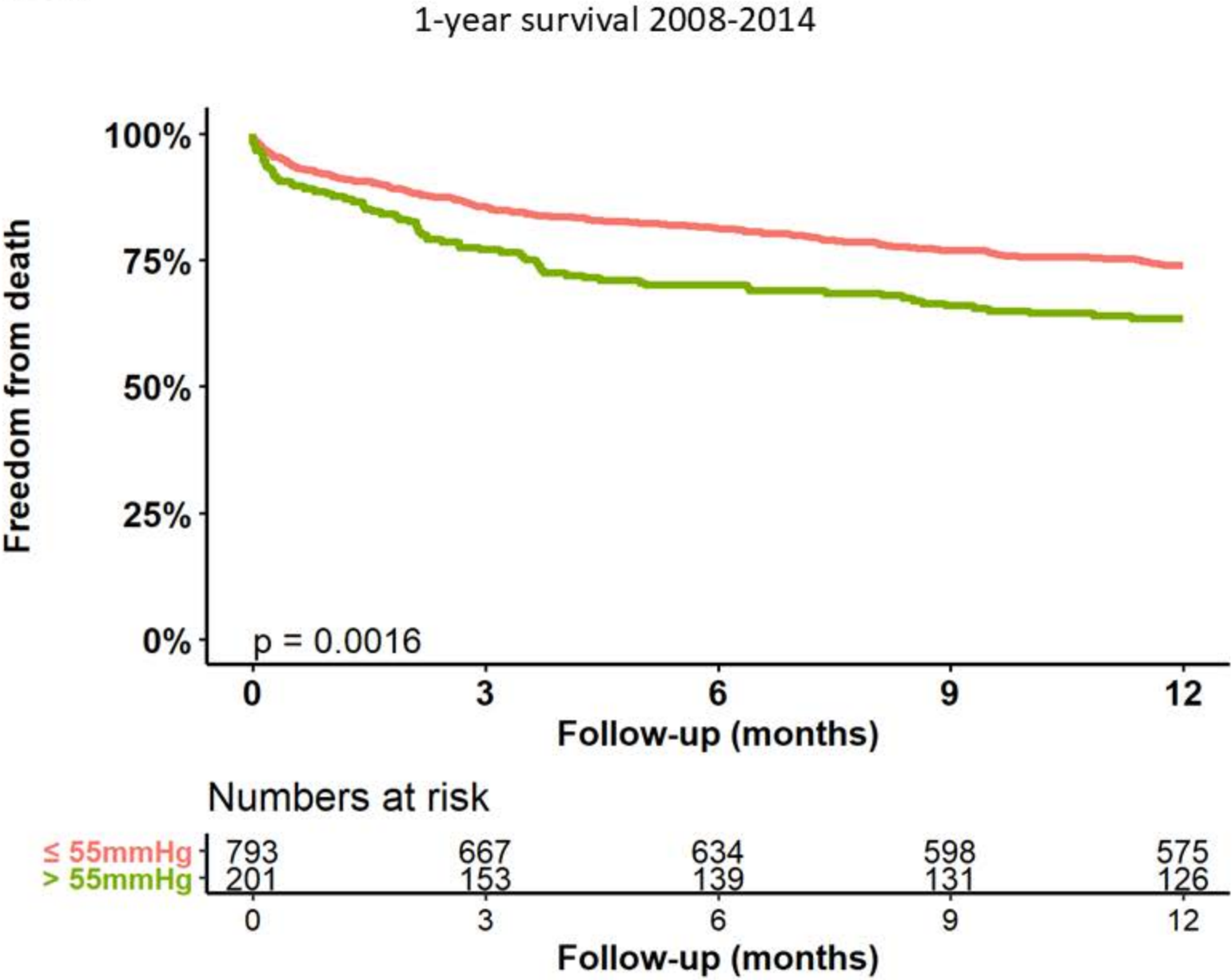

Supplementary Figure 3b.

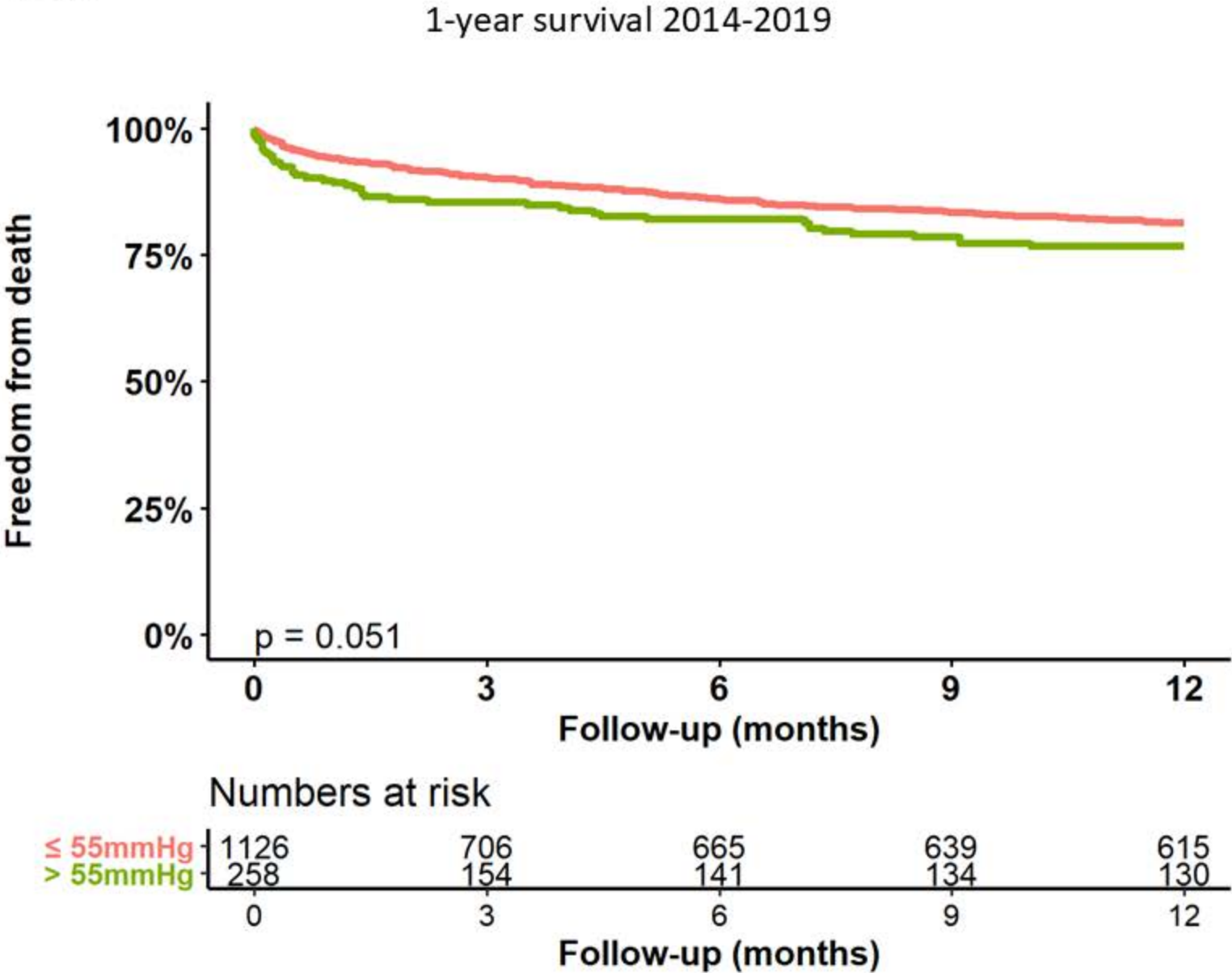

Supplementary Figure 3c.

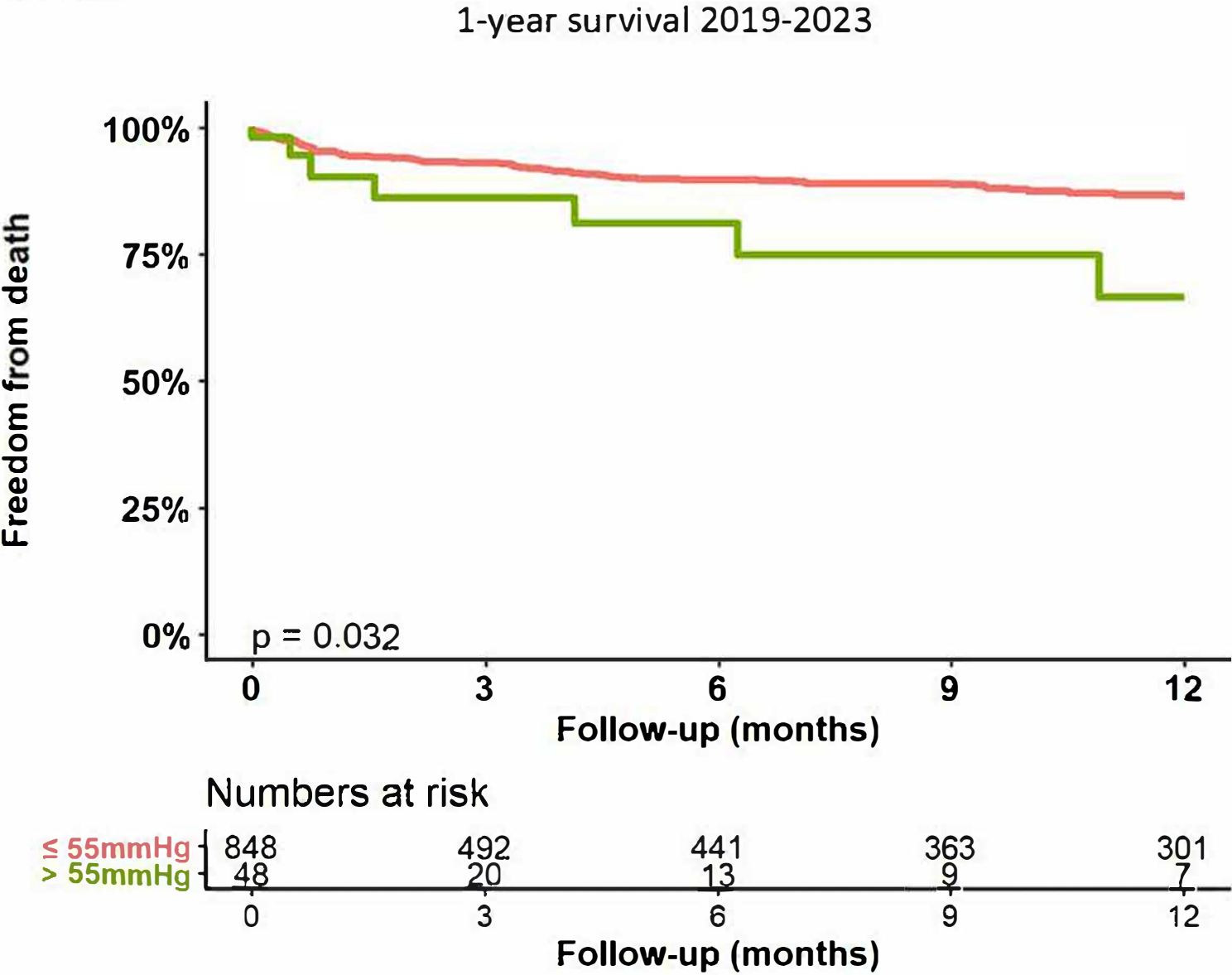

Supplement: Supplementary file 3 [file Image3.pdf]
